# Trans-Scleral Plugs Fixated FIL SSF IOL: A Review of the Literature and Comparison with Other Secondary IOL Implants

**DOI:** 10.3390/jcm12051994

**Published:** 2023-03-02

**Authors:** Raffaele Raimondi, Tania Sorrentino, Raphael Kilian, Yash Verma, Francesco Paolo De Rosa, Giuseppe Cancian, Panos Tsoutsanis, Giovanni Fossati, Davide Allegrini, Mario R. Romano

**Affiliations:** 1 Department of Biomedical Sciences, Humanitas University, Via Rita Levi Montalcini 4, 20090 Pieve Emanuele, Italy; 2 Ophthalmic Unit, Department of Neurosciences, Biomedicine and Movement Sciences, University of Verona, 37129 Verona, Italy; 3 Eye Center, Humanitas Gavazzeni-Castelli, 24128 Bergamo, Italy

**Keywords:** Carlevale lens, secondary implant, scleral fixated intraocular lens, suture less fixation

## Abstract

**Purpose.** To revise the current literature on FIL SSF (Carlevale) intraocular lens, previously known as Carlevale lens, and to compare their outcomes with those from other secondary IOL implants. **Methods.** We performed a peer review of the literature regarding FIL SSF IOLs until April 2021 and analyzed the results only of articles with a minimum of 25 cases and a follow-up of at least 6 months. The searches yielded 36 citations, 11 of which were abstracts of meeting presentations that were not included in the analysis because of their limited data. The authors reviewed 25 abstracts and selected six articles of possible clinical relevance to review in full text. Of these, four were considered to be sufficiently clinically relevant. Particularly, we extrapolated data regarding the pre- and postoperative best corrected visual acuities (BCVA) and the complications related to the procedure. The complication rates were then compared with those from a recently published Ophthalmic Technology Assessment by the American Academy of Ophthalmology (AAO) on secondary IOL implants. **Results.** Four studies with a total of 333 cases were included for results analysis. The BCVA improved in all cases after surgery, as expected. Cystoid macular edema (CME) and increased intraocular pressure were the most common complications, with an incidence of up to 7.4% and 16.5%, respectively. Other IOL types from the AAO report included anterior chamber IOLs, iris fixation IOLs, sutured iris fixation IOLs, sutured scleral fixation IOLs, and sutureless scleral fixation IOLs. There was no statistically significant difference in the rates of postoperative CME (*p* = 0.20), and vitreous hemorrhage (*p* = 0.89) between other secondary implants and the FIL SSF IOL, whereas the rate of retinal detachment was significantly less with FIL SSF IOLs (*p* = 0.04). **Conclusion.** The results of our study suggest the implantation of FIL SSF IOLs is an effective and safe surgical strategy in cases where there is a lack of capsular support. In fact, their outcomes seem to be comparable to those obtained with the other available secondary IOL implants. According to published literature, the FIL SSF (Carlevale) IOL provides favorable functional results with a low rate of postoperative complications.

## 1. Introduction

**Figure 1:**
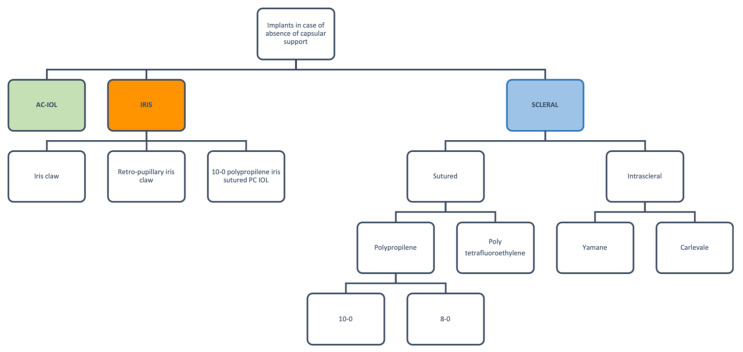
Schematic representation of implant options in the absence of capsular support.

Posterior capsular rupture is one of the most common complications of cataract surgery [1]. Different secondary intraocular lens (IOL) implants have been proposed in the absence of a capsular support (Figure 1), and even though open-loop anterior chamber IOLs are the only FDA-approved ones, several other options are also available. However, the question of which of these options produces the best outcomes remains a topic of debate.

Recently, a new trans-scleral plug IOL (FIL SSF) was introduced on the market and has shown to provide excellent clinical results [2].

### 1.1. Anterior Chamber IOLs

Flexible one-piece polymethyl methacrylate (PMMA) open-loop anterior chamber IOLs (ACIOLs) are foldable lenses whose haptics are placed against the scleral spur. Despite the short surgical time and the sutureless technique, the implantation site of such IOLs presents several drawbacks [3]. First, these lenses can lead to corneal endothelial damage, increasing the risk of endothelial decompensation [4]. Second, the trabecular meshwork might also be injured, and the IOL itself might rub against the iris, causing chronic inflammation, IOP increase, and an uveitis-glaucoma-hyphema syndrome. This is more frequent in case the ACIOL is not properly sized or flexible. Overall, these complications occur more commonly with close-looped AC IOLs rather than with open-looped ones [5,6]. Surgeons should refrain from using these implants on younger patients or patients who have a history of uveitis. Additionally, one-piece ACIOLs are not suitable for patients with shallow ACs, irideal abnormalities, or endothelial dysfunction [7].

### 1.2. Iris Fixation IOLs

There are two types of iris-fixated lenses: the iris-claw lens (EU Artisan; USA Verisyse Ophtec, Groningen, The Netherlands) and iris-sutured IOLs (the former being an evolution of the Worst iris-claw lens). The iris-claw lens is a PMMA lens whose haptics form clips grasping the irideal tissue at the midperipheral portion of the iris, in order not to interfere with the normal physiology of the iris (i.e., pupillary dilation and constriction) or that of the angular structures [6]. As for ACIOLs, the short surgical time and the independence from sutures are important advantages; however, they may still lead to possible complications such as endothelial cell loss, iris atrophy, lens dislocation, pupillary distortion, and macular edema [8]. Iris-claw lenses can be placed either at a pre-pupillary or at a retro-pupillary level. In addition to their location, these two do not show marked differences with regards to optical outcomes; however, retro-pupillary claw lenses have been noted to present a lower rate of endothelial cell loss [8].

Iris-sutured IOLs, on the other hand, can also be placed in the posterior chamber with the help of 10-0 polypropylene sutures securing their haptics on the iris. However, due to the fragility of the iris, some degree of lenticular mobility is always present, thus predisposing to pseudo-phacodonesis and tilting of the IOL. In addition, this procedure is much more cumbersome than the insertion of an open-loop AC IOL, especially when a limbal approach is used (i.e., when the procedure is not coupled with penetrating keratoplasty) [5,9].

### 1.3. Scleral Fixation IOLs

Scleral-fixated IOLs (SFIOLs) can be positioned either via transscleral sutures or with suture-independent techniques.

Sutured SFIOLs require either polypropylene (Prolene) or polytetrafluoroethylene (Gore-Tex) suture material to be passed through specific holes in the IOL haptics and eventually fixed at the sclera. Given the high risk of knot erosion that characterizes 10-0 Prolene sutures [10], which in turn increases the risk of endophthalmitis [10], 8-0 Prolene or Gore-Tex sutures are generally preferred.

Due to the risks associated with sutures, researchers have investigated sutureless methods for intrascleral fixation. The Yamane technique and the fibrin glue technique by Agarwal are among the most well-known, both of which use 3-piece IOLs off-label. The Yamane technique involves creating two angled sclerotomies at the end of two lamellar scleral dissections. The IOL’s haptics are then brought outside onto the sclera, and their ends are cauterized using an ophthalmic cautery device to create a flange for each haptic. Finally, the haptics are secured in position within scleral tunnels created using a needle [11].

Agarwal’s technique, on the other hand, is based on the generation of two partial-thickness scleral flaps, below which the haptics are externalized and eventually fixed with the help of fibrin glue [12].

Since the introduction of Yamane and Agarwal’s techniques, a great deal of interest was raised in devising an approved sutureless SFIOL. This resulted in the conception of the FIL SSF IOL (Soleko, Rome, Italy), a foldable, one-piece, acrylic lens with 25% H_2_O and a UV filter. This IOL presents with two transcleral T-shaped plugs/haptics anchoring the lens to the sclera without the use of sutures and without requiring any adjustments in the positioning of the haptics [2,13]. FIL SSF IOLs minimize IOL tilting though a T-shaped harpoon and four scleral sulcus counterpressure points; however, in order to obtain a good IOL centration, a symmetrical positioning of the sclerotomies is mandatory [2,14,15].

The purpose of our study was to revise published results of this trans-scleral plug implant and to compare them with those highlighted in a recently published American Academy of Ophthalmology (AAO) ophthalmic technology assessment on various secondary IOL implants [7]. This review reports published outcomes of FIL SSF IOL and compares complication rates with the AAO ophthalmology technology assessment which does not include this IOL.

## 2. Methods

The peer-reviewed literature on FIL SSF IOLs was analyzed, and all articles regarding these were selected until April 2021. The research was conducted on Medline, on CENTRAL, and on the World Health Organization (WHO) International Clinical Trials Registry Platform (ICTRP) and was limited to studies published in English. The search strategy used the following MeSH terms and text words: sutureless scleral fixation, FIL SSF, sutureless scleral lens, and FIL SSF IOL.

The initial search yielded 36 citations, 11 of which were abstracts of meeting presentations that were not included in the analysis because of their limited data. The authors reviewed 25 abstracts and selected six articles of possible clinical relevance to review in full text. Of these, four were considered to be sufficiently clinically relevant. According to the AAO criteria [9] and to avoid the biases of smaller studies, only reports with at least 25 adult participants and a minimum 6-month mean follow-up were included in this analysis.

The panel methodologist (R.R.) assessed and assigned each study with a level of evidence rating according to the American Academy of Ophthalmology’s guidelines and using the rating scale developed by the British Centre for Evidence-Based Medicine. Level I articles were well-designed and well-conducted randomized clinical trials, level II was assigned to well-designed and well-conducted cohort and case-control studies, and level III to case series. Since no articles satisfied the level I-level III evidence requirements, all articles were rated as having level II evidence.

Results from the analyzed studies were then compared to those extrapolated from a recently published ophthalmic technology assessment by the American Academy of Ophthalmology on intraocular lenses, in the absence of zonular support. Particularly, the complications that were compared, were only those for which every analyzed study had published results.

### Statistical Analysis

To carry out the statistical analysis, we used the STATA/IC 16 software, and all data were expressed as mean—standard deviation. A one-way analysis of variance (ANOVA) was used to investigate any statistically significant differences between the means. The differences were considered statistically significant if *p* value was <0.05.

## 3. Results

### 3.1. Visual Outcomes

A recent report by Barca et al. on 32 eyes implanted with a FIL SSF IOL found an increase in the mean BCVA from 0.46 ± 0.29 logMAR preoperatively to 0.13 ± 0.12 logMAR 8 months after the procedure (*p* < 0.05). In another study on 78 patients, Rossi et al. also found a significant increase in BCVA, i.e., from 0.86 ± 0.56 logMAR to 0.38 ± 0.42 logMAR at 6 months after scleral fixation of the IOL, (*p* < 0.001). Similar results on a much larger cohort of patients were reported by Georgalas et al. In their study, they followed up on 169 eyes for a mean 9-month period and reported an increase in mean BCVA from 0.58 ± 0.49 logMAR to 0.09 ± 0.1 logMAR, (*p* = 0.0001). In 2021, Vaiano et al. investigated the visual outcomes associated with FIL SSF IOL implantation in 54 eyes and found an improvement from 0.93 ± 0.61 logMAR to 0.42 ± 0.34 (logMAR), 0.42 ± 0.37 (logMAR), and 0.38 ± 0.38 (logMAR), respectively, at 3, 6, and 12 months from the surgery (Table 1).

### 3.2. Complications

The most frequent complication reported among the analyzed studies was increased intraocular pressure (IOP), followed by cystoid macular edema (CME). The mean endothelial cell loss was analyzed only by Barca et al. and was found to have decreased after the surgery (i.e., from 2307 ± 406 to 2208 ± 372, *p* < 0.01). The exact distribution of the complications among each group of study can be found in Table 2. Notably, no cases of lens tilt or lens decentration were reported. In addition, none of the studies reported the need for postoperative IOL explantation for any reason.

### 3.3. Comparison with Other Secondary IOL Implants

The weighted data coming from our review and those coming from the AAO report on complications from secondary IOL implants are shown in Table 3, whereas the raw data from each single study can be found in Table 4, Table 5 and Table 6.

Given that the cohorts of patients in the different studies might have had heterogeneous basal conditions determining the need for a secondary IOL implant, we felt that the comparison between the final BCVAs amongst the various studies might have been biased and decided to only compare the complication rates.

**Figure 2:**
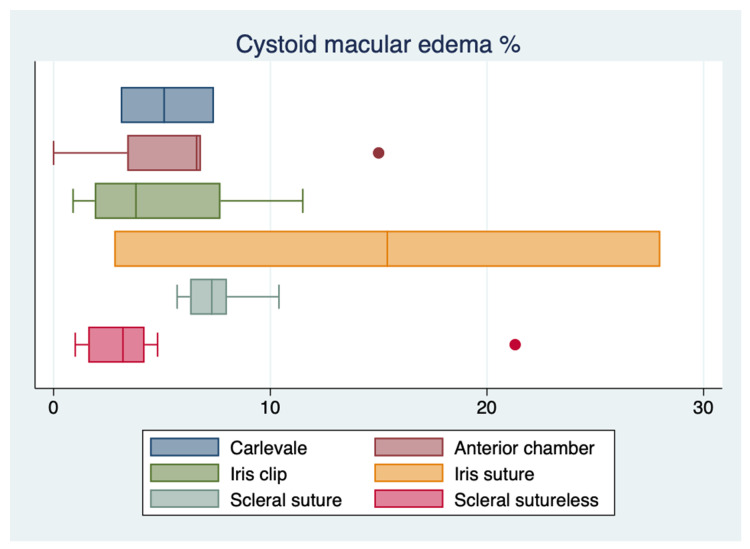
Boxplot of cystoid macular edema reported incidences.

**Figure 3:**
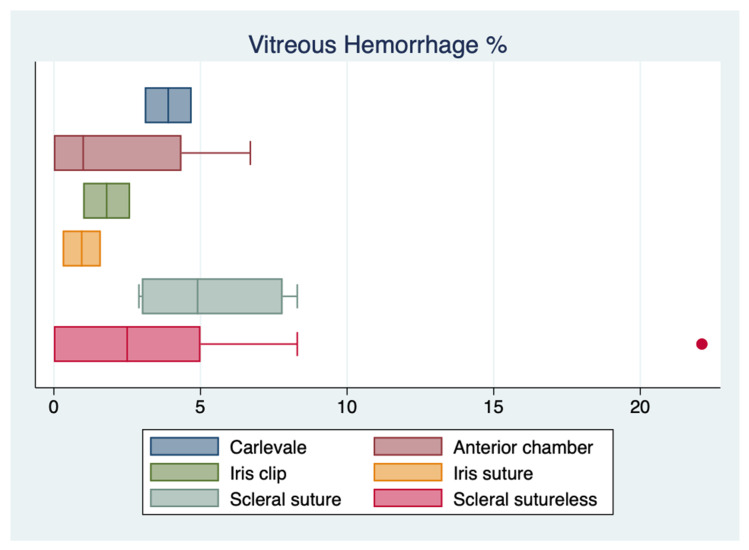
Boxplot of vitreous hemorrhage reported incidences.

**Figure 4:**
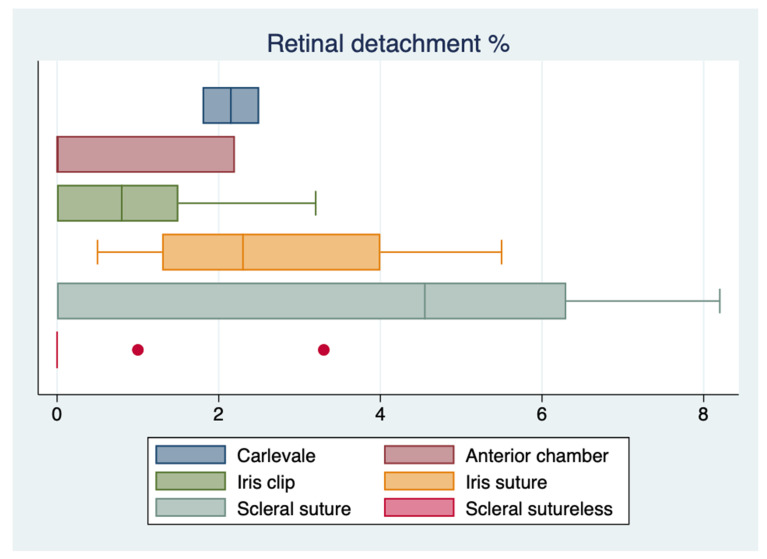
Boxplot of retinal detachment reported incidences.

The results of the one-way ANOVA for the most extensively reported complications showed no statistically significant difference in the rates of CME (*p* = 0.20) and vitreous hemorrhage (*p* = 0.89) between other secondary implants and the FIL SSF IOL, whereas there was a statistically significant difference in the rate of retinal detachment (*p* = 0.04). However, the sample size was too small to perform a Wilcoxon rank-sum test and compare the means between the different techniques. (Figure 2, Figure 3 and Figure 4).

## 4. Discussion

To our knowledge, the results of this review summarize for the first time the visual outcomes and complications of this new scleral-fixated IOL. Overall, the implantation of the FIL SSF IOL seems to be a safe and effective technique. In addition, similar outcomes are reported by different authors, which suggests a good reproducibility of the surgical technique.

When looking at the other secondary IOL implantation techniques included in the AAO technology assessment, several points need to be highlighted. Comparing FIL SSF IOLs with 10-0 propylene scleral-sutured posterior chamber IOLs, the former show a lower incidence of cystoid macular edema and of retinal detachment, although not statistically significant. In fact, for 10-0 propylene scleral-sutured posterior chamber IOLs, the AAO technology assessment reported a rate of macular edema and retinal detachment that varied between 5.7 and 10.4% and between 0 and 8.2%, respectively. Indeed, the surgical technique needed to implant a FIL SSF might be easier than that implying a scleral sutured IOL, so that the surgeon causes less damage to the posterior chamber structures, thus reducing the complication rate. Moreover, the FIL SSF IOL allows firm intrascleral fixation of haptics, granting the possibility of safe, extensive scleral indentation to detect any possible peripheral retinal tear. This may explain the low retinal detachment rates reported.

Intrascleral haptic fixation posterior chamber IOLs are another type of scleral-sutured lens that has been extensively studied in the literature. In most published studies, the rate of the main complications is comparable to that of FIL SSF IOLs, with the exception of the series published by Todorich et al. [41], which reported a 21.3% incidence of cystoid macular edema.

The 10-0 polypropylene iris-sutured posterior chamber IOLs, on the other hand, were reported to induce cystoid macular edema in up to 28% of cases [40], and to have a variable retinal detachment rate between 0.5 and 5.5%.

Unlike the above-described lenses, iris-claw IOLs are held in position by the fixation of their haptics to the iris. Even though some studies reported no incidence of cystoid macular edema, others have shown this complication occurring in up to 11.5% of patients. Moreover, postoperative uveitis has been described in a significant percentage of patients, i.e., 7.7% [18], whereas this complication has never been reported in studies regarding FIL SSF IOLs.

Along with a pretty high incidence of postoperative glaucoma (up to 16.7%) and cystoid macular edema (up to 15%), anterior chamber IOLs have also been described as being considerably affected by chronic uveitis (up to 20%). The results of our study show that these three major complications are less common when considering FIL SSF IOLs.

Nonetheless, the lack of comparative studies makes it difficult to compare the rate of complications of the FIL SSF IOLs with that of other IOL types implanted in the absence of zonular support. Despite lacking some precision (i.e., different studies might have had cohorts of patients with different basal conditions), in this study we proposed a simple and effective way of comparing the results of different secondary IOL implants. By analyzing the incidence of the major characteristic complications, it is evident that the FIL SSF IOL showed a good safety profile, which is comparable, and in certain instances superior, to other IOLs implanted in the absence of zonular support [51].

Indeed, the FIL SSF IOL is a foldable IOL, composed of 25% H_2_O acrylic and is designed to have flexible sclero-corneal plugs at the end of its two haptics, allowing it to be implanted posterior to the iris and to attach to the sclera in a sutureless fashion. It has an optic diameter of 6.5 mm, a total length of 13.2 mm, and is available in IOL powers ranging from −5.0 to +35.0 diopters [52].

The FIL SSF lens is a remarkable invention with a number of advantages that surpass the previously described implants. Specifically, it is designed to minimize lens tilting and associated multiple aberrations through its T-shaped harpoon and four scleral sulcus counterpressure points. This is in contrast to other types of lenses such as ACIOL, IFIOL, or SFIOL, which commonly require sutures for fixation, and subsequently expose patients to significant postoperative risks. These risks include corneal decompensation, erosion into angle structures, pupillary block, suprachoroidal or vitreous hemorrhage, retinal detachment, lens tilt or dislocation, and suture erosion, among others.

However, the FIL SSF lens has been shown to effectively reduce these risks by employing a sutureless scleral fixation (SSF) procedure during implantation, which not only ensures a simplified surgical process but also reduces intraoperative time. The innovative design and technology of the FIL SSF lens have revolutionized the field of secondary implants, offering a safer and more efficient alternative to traditional IOL implants. Furthermore, even though no previous study has ever evaluated the impact of this factor on the final BCVA, unlike other IOLs, where manipulation of the haptics is needed to achieve IOL centration, symmetrical positioning of the sclerotomies allows the FIL SSF IOLs to be placed in a secured, centrally aligned position, thus avoiding possible post-operative visual aberrations [2,14,15]. Moreover, it is important to highlight that the FIL SSF IOL is the only labeled IOL for scleral fixation, while other techniques use lenses labeled for capsular bag implants.

Future research adopting computational simulation can be useful to calculate and predict refractive outcomes in complicated cases; this technology is widely applied with success in other fields and can potentially boost results [53].

Among the drawbacks of the FIL SSF IOL is its hydrophilic nature. In fact, this may lead to IOL opacification if air or a gaseous tamponading agent is being used, or late opacification after implantation, which, on the contrary, is very rare with hydrophobic lenses [54]. Accordingly, for the labeling company, new research is currently in place to switch to a hydrophobic FIL SSF IOL. Lastly, in the analyzed literature, it was not possible to retrieve the distance from the limbus and the covering technique of the T-shape haptic that may impact the complication rate.

This review has several limitations. Firstly, in order to compare the outcomes with the AAO technology assessment, some studies were not included in this analysis, which limited the number of cases analyzed. Secondly, we were not able to compare functional results since improvement in BCVA is highly dependent on corneal and retinal status, which was not reported in the analyzed studies.

## 5. Conclusions

Numerous published studies have demonstrated that the FIL SSF IOL represents a secure and reliable device with a low incidence of complications. Moreover, its performance and efficacy appear to be on par with other available techniques currently in use. However, despite these promising findings, it is imperative to conduct further research in the form of prospective comparative studies in order to fortify the scientific evidence supporting the effectiveness of this technique.

## Figures and Tables

**Table 1:** Weighted mean preoperative and postoperative best-corrected visual acuity by Carlevale lens technique.

Authors	Total N. of Eyes	Mean Follow-Up	BCVA (logMAR)	
			Preoperative	Postoperative
Barca et al. [2]	32	8	0.46 ± 0.29	0.13 ± 0.12
Rossi et al. [14]	78	6	0.86 ± 0.56	0.38 ± 0.42
Georgalas et al. [13]	169	9	0.58 ± 0.49	0.09 ± 0.1
Vaiano et al. [16]	54	12	0.42 ± 0.34	0.38 ± 0.38

**Table 2:** Complications rates in the analyzed publications.

Authors	Cystoid Macular Edema	IOP Increase	Reverse Pupillary Block	Vitreous Hemorrhage	Retinal Tears	Retinal Detachment	Corneal Decompensation	Haptic Exposure
Barca et al. [2]	1 (3.1%)	1 (3.1%)	2 (6.2%)	1 (3.1%)				
Rossi et al. [14]	4 (5.1%)	2 (2.5%)			2 (2.5%)	2 (2.5%)	1 (1.3%)	
Georgalas et al. [13]		28 (16.5%)		8 (4.7%)				
Vaiano et al. [16]	4 (7.4%)					1(1.8%)		2 (3.7%)

**Table 3:** Intraocular lens weighted complication means.

Lens Type	Total N. of Eyes	CME	Vitreous Haemorrhage %	Retinal Detachment %
Carlevale	333	5.4	4.4	2.2
Anterior chamber IOL	311	7.3	2.2	0.9
Iris fixation, anterior	254	4.2	1.2	0.4
Iris fixation, posterior	629	2.8	0.3	0.7
Iris fixation suture	639	16.2	0.7	2.0
Scleral fixation suture	1163	4.8	4.3	2.2
Scleral fixation sutureless	1331	4.5	3.4	0.5

**Table 4:** Cystoid macular edema incidence of complications of different implants.

CME Carlevale	CME Angle	CME Iris Clip	CME Iris Suture	CME Scleral Suture	CME Sclera Sutureless
3.1 [2]	6.6 [17]	7.7 [18]	28 [19]	7.3 [20]	1.6 [21]
5.1 [14]	6.8 [22]	3.1 [23]	2.8 [24]	6.3 [25]	1.4 [26]
7.4 [16]	15 [27]	0.9 [28]		5.7 [29]	4.8 [26]
	0 [30]	4.5 [31]		10.4 [32]	4 [33]
	3.4 [34]	11.5 [35]		8 [36]	2.9 [37]
		1.9 [38]			4.2 [39]
					3 [40]
					1 [11]
					3.3 [38]
					2. [38]
					21.3 [41]

**Table 5:** Vitreous hemorrhage incidence of complications of different implants.

VH Carlevale	VH Angle	VH Iris Clip	VH Iris Suture	VH Scleral Suture	VH Sclera Sutureless
3.1 [2]	0 [17]	2.6 [18]	1.6 [42]	4.8 [43]	3.2 [21]
4.7 [13]	0 [22]	1 [38]	0.3 [19]	3 [44]	0 [33]
	2.2 [30]			2.9 [29]	0 [37]
	6.7 [45]			8.3 [32]	8.3 [39]
				7.8 [46]	0 [40]
				5 [36]	5 [11]
					0 [38]
					2.5 [38]
					22.1 [41]

**Table 6:** Retinal detachment incidence of complications of different implants.

RD Carlevale	RD Angle	RD Iris Clip	RD Iris Suture	RD Scleral Suture	RD Sclera Sutureless
2.5 [14]	0 [17]	0.8 [18]	0.5 [42]	8.2 [47]	1 [26]
1.8 [17]	0 [22]	0 [48]	2.5 [19]	6.3 [43]	0 [26]
	0 [27]	3.2 [49]	2.1 [24]	4.9 [20]	0 [33]
	2.2 [30]	0.3 [28]	5.5 [31]	4.2 [32]	0 [37]
	2.2 [45]	1.5 [31]			0 [39]
		0 [50]			0 [40]
		1 [38]			0 [11]
					3.3 [38]
					0 [38]

## Data Availability

Data generated or analyzed during this study are included in this article. Further enquiries can be directed to the corresponding author.

## References

[1] Chen M., LaMattina K., Patrianakos T., Dwarakanathan S. (2014). Complication rate of posterior capsule rupture with vitreous loss during phacoemulsification at a Hawaiian cataract surgical center: A clinical audit. Clin. Ophthalmol..

[2] Barca F., Caporossi T., de Angelis L., Giansanti F., Savastano A., Di Leo L., Rizzo S. (2020). Trans-scleral plugs fixated IOL: A new paradigm for sutureless scleral fixation. J. Cataract. Refract. Surg..

[3] Lyle W.A., Jin J.-C. (1993). Secondary Intraocular Lens Implantation: Anterior Chamber vs. Posterior Chamber Lenses. Ophthalmic Surg. Lasers Imaging Retin..

[4] Ravalico G., Botteri E., Baccara F. (2003). Long-term endothelial changes after implantation of anterior chamber intraocular lenses in cataract surgery. J. Cataract. Refract. Surg..

[5] Dick H.B., Augustin A.J. (2001). Lens implant selection with absence of capsular support. Curr. Opin. Ophthalmol..

[6] Smith P.W., Wong S.K., Stark W.J., Gottsch J.D., Terry A.C., Bonham R.D. (1987). Complications of Semiflexible, Closed-Loop Anterior Chamber Intraocular Lenses. Arch. Ophthalmol..

[7] Shen J.F., Deng S., Hammersmith K.M., Kuo A.N., Li J.Y., Weikert M.P., Shtein R.M. (2020). Intraocular Lens Implantation in the Absence of Zonular Support: An Outcomes and Safety Update: A Report by the American Academy of Ophthalmology. Ophthalmology.

[8] Peralba R.T., Lamas-Francis D., Sarandeses-Diez T., Martínez-Pérez L., Rodríguez-Ares T. (2018). Iris-claw intraocular lens for aphakia: Can location influence the final outcomes?. J. Cataract. Refract. Surg..

[9] Wagoner M.D., Cox T.A., Ariyasu R.G., Jacobs D.S., Karp C.L. (2003). Intraocular lens implantation in the absence of capsular support: A report by the American Academy of Ophthalmology. Ophthalmology.

[10] Kim J., Kinyoun J.L., Saperstein D.A., Porter S.L. (2003). Subluxation of transscleral sutured posterior chamber intraocular lens (TSIOL). Am. J. Ophthalmol..

[11] Yamane S., Sato S., Maruyama-Inoue M., Kadonosono K. (2017). Flanged Intrascleral Intraocular Lens Fixation with Double-Needle Technique. Ophthalmology.

[12] Agarwal A., Kumar D.A., Jacob S., Baid C., Agarwal A., Srinivasan S. (2008). Fibrin glue–assisted sutureless posterior chamber intraocular lens implantation in eyes with deficient posterior capsules. J. Cataract. Refract. Surg..

[13] Georgalas I., Spyropoulos D., Gotzaridis S., Papakonstantinou E., Kandarakis S., Kanakis M., Karamaounas A., Petrou P. (2021). Scleral fixation of Carlevale intraocular lens: A new tool in correcting aphakia with no capsular support. Eur. J. Ophthalmol..

[14] Rossi T., Iannetta D., Romano V., Carlevale C., Forlini M., Telani S., Imburgia A., Mularoni A., Fontana L., Ripandelli G. (2020). A novel intraocular lens designed for sutureless scleral fixation: Surgical series. Graefe’s Arch. Clin. Exp. Ophthalmol..

[15] Petrelli M., Schmutz L., Gkaragkani E., Droutsas K., Kymionis G.D. (2020). Simultaneous penetrating keratoplasty and implantation of a new scleral-fixated, sutureless, posterior chamber intraocular lens (Soleko, Carlevale): A novel technique. Cornea.

[16] Vaiano A.S., Hoffer K.J., Greco A., Greco A., D’Amico G., Pasqualitto V., Carlevale C., Savini G. (2021). Long-term Outcomes and Complications of the New Carlevale Sutureless Scleral Fixation Posterior Chamber IOL. J. Refract. Surg..

[17] Dadeya S., Kamlesh P.K.S. (2003). Secondary Intraocular Lens (IOL) Implantation: Anterior Chamber versus Scleral Fixation Long-Term Comparative Evaluation. Eur. J. Ophthalmol..

[18] De Silva S.R., Arun K., Anandan M., Glover N., Patel C.K., Rosen P. (2011). Iris-claw intraocular lenses to correct aphakia in the absence of capsule support. J. Cataract. Refract. Surg..

[19] Farjo A.A., Rhee D.J., Soong H.K., Meyer R.F., Sugar A. (2004). Iris-Sutured Posterior Chamber Intraocular Lens Implantation During Penetrating Keratoplasty. Cornea.

[20] McAllister A.S., Hirst L.W. (2011). Visual outcomes and complications of scleral-fixated posterior chamber intraocular lenses. J. Cataract. Refract. Surg..

[21] Scharioth G.B., Prasad S., Georgalas I., Tataru C., Pavlidis M. (2010). Intermediate results of sutureless intrascleral posterior chamber intraocular lens fixation. J. Cataract. Refract. Surg..

[22] Evereklioglu C., Er H., Bekir N.A., Borazan M., Zorlu F. (2003). Comparison of secondary implantation of flexible open-loop anterior chamber and scleral-fixated posterior chamber intraocular lenses. J. Cataract. Refract. Surg..

[23] Güell J.L., Verdaguer P., Elies D., Gris O., Manero F., Mateu-Figueras G., Morral M. (2014). Secondary iris-claw anterior chamber lens implantation in patients with aphakia without capsular support. Br. J. Ophthalmol..

[24] Condon G.P., Masket S., Kranemann C., Crandall A.S., Ahmed I.I.K. (2007). Small-Incision Iris Fixation of Foldable Intraocular Lenses in the Absence of Capsule Support. Ophthalmology.

[25] Burcu A., Yalniz-Akkaya Z., Abay I., Acar M.A., Ornek F. (2013). Scleral-Fixated Posterior Chamber Intraocular Lens Implantation in Pediatric and Adult Patients. Semin. Ophthalmol..

[26] Kumar D.A., Agarwal A. (2013). Glued intraocular lens: A major review on surgical technique and results. Curr. Opin. Ophthalmol..

[27] Donaldson K.E., Gorscak J.J., Budenz D.L., Feuer W.J., Benz M.S., Forster R.K. (2005). Anterior chamber and sutured posterior chamber intraocular lenses in eyes with poor capsular support. J. Cataract. Refract. Surg..

[28] Forlini M., Soliman W., Bratu A., Rossini P., Cavallini G.M., Forlini C. (2015). Long-term follow-up of retropupillary iris-claw intraocular lens implantation: A retrospective analysis. BMC Ophthalmol..

[29] Mahmood S.A., Zafar S., Shakir M., Rizvi S.F. (2014). Visual acuity after trans-scleral sutured posterior chamber intraocular lens. J. Coll. Physicians Surg. Pak..

[30] Kwong Y.Y., Yuen H.K., Lam R.F., Lee V.Y., Rao S.K., Lam D.S. (2007). Comparison of Outcomes of Primary Scleral-Fixated versus Primary Anterior Chamber Intraocular Lens Implantation in Complicated Cataract Surgeries. Ophthalmology.

[31] Faria M.Y., Ferreira N.P., Pinto J.M., Cordeiro-Sousa D., Cardoso-Leal I., Neto E., Marques-Neves C. (2016). Retropupillary iris claw intraocular lens implantation in aphakia for dislocated intraocular lens. Int. Med. Case Rep. J..

[32] Long C., Wei Y., Yuan Z., Zhang Z., Lin X., Liu B. (2015). Modified technique for transscleral fixation of posterior chamber intraocular lenses. BMC Ophthalmol..

[33] Narang P., Narang S. (2013). Glue-assisted intrascleral fixation of posterior chamber intraocular lens. Indian J. Ophthalmol..

[34] Jamwal R. (2018). Bioavailable curcumin formulations: A review of pharmacokinetic studies in healthy volunteers. J. Integr. Med..

[35] Kumar K.V., Jayamadhury G., Potti S., Kumar R.M., Mishra K.D., Nambula S.R. (2016). Retropupillary fixation of iris-claw lens in visual rehabilitation of aphakic eyes. Indian J. Ophthalmol..

[36] Yeung L., Wang N.-K., Wu W.-C., Chen K.-J. (2018). Combined 23-gauge transconjunctival vitrectomy and scleral fixation of intraocular lens without conjunctival dissection in managing lens complications. BMC Ophthalmol..

[37] Yamane S., Inoue M., Arakawa A., Kadonosono K. (2014). Sutureless 27-Gauge Needle–Guided Intrascleral Intraocular Lens Implantation with Lamellar Scleral Dissection. Ophthalmology.

[38] Kelkar A., Shah R., Vasavda V., Kelkar J., Kelkar S. (2017). Primary iris claw IOL retrofixation with intravitreal triamcinolone acetonide in cases of inadequate capsular support. Int. Ophthalmol..

[39] Kawaji T., Sato T., Tanihara H. (2016). Sutureless intrascleral intraocular lens fixation with lamellar dissection of scleral tunnel. Clin. Ophthalmol..

[40] Zhang Y., He F., Jiang J., Li Q., Wang Z. (2017). Modified technique for intrascleral fixation of posterior chamber intraocular lens without scleral flaps. J. Cataract. Refract. Surg..

[41] Todorich B., Stem M.S., Kooragayala K., Thanos A., Faia L.J., Williams G.A., Hassan T.S., Woodward M.A., Wolfe J.D. (2018). Structural analysis and comprehensive surgical outcomes of the sutureless intrascleral fixation of secondary intraocular lenses in human eyes. Retina.

[42] Akpek E.K., Altan-Yaycioglu R., Karadayi K., Christen W., Stark W.J. (2003). Long-term outcomes of combined penetrating keratoplasty with iris-sutured intraocular lens implantation. Ophthalmology.

[43] Bading G., Hillenkamp J., Sachs H.G., Gabel V.-P., Framme C. (2007). Long-term Safety and Functional Outcome of Combined Pars Plana Vitrectomy and Scleral-Fixated Sutured Posterior Chamber Lens Implantation. Am. J. Ophthalmol..

[44] Kim S.J., Lee S.J., Park C.H., Jung G.Y., Park S.H. (2009). Long-Term Stability and Visual Outcomes of a Single-Piece, Foldable, Acrylic Intraocular Lens for Scleral Fixation. Retina.

[45] Chan T., Lam J.K., Jhanji V., Li E.Y. (2015). Comparison of Outcomes of Primary Anterior Chamber Versus Secondary Scleral-Fixated Intraocular Lens Implantation in Complicated Cataract Surgeries. Am. J. Ophthalmol..

[46] Kang D.J., Kim H.K. (2016). Clinical analysis of the factors contributing to pupillary optic capture after transscleral fixation of posterior chamber intraocular lens for dislocated intraocular lens. J. Cataract. Refract. Surg..

[47] Vote B.J., Tranos P., Bunce C., Charteris D.G., Da Cruz L. (2006). Long-Term Outcome of Combined Pars Plana Vitrectomy and Scleral Fixated Sutured Posterior Chamber Intraocular Lens Implantation. Am. J. Ophthalmol..

[48] Mohammad-Rabie H., Malekifar P., Esfandiari H. (2016). Visual Outcomes after Primary Iris Claw Artisan Intraocular Lens Implantation during Complicated Cataract Surgery. Semin. Ophthalmol..

[49] Schallenberg M., Dekowski D., Hahn A., Laube T., Steuhl K.-P., Meller D. (2013). Aphakia correction with retropupillary fixated iris-claw lens (Artisan)—Long-term results. Clin. Ophthalmol..

[50] Choragiewicz T., Rejdak R., Grzybowski A., Nowomiejska K., Moneta-Wielgoś J., Ozimek M., Jünemann A.G.M. (2016). Outcomes of Sutureless Iris-Claw Lens Implantation. J. Ophthalmol..

[51] Schnurrbusch U.E.K., Welt K., Horn L.-C., Wiedemann P., Wolf S. (2001). Histological findings of surgically excised choroidal neovascular membranes after photodynamic therapy. Br. J. Ophthalmol..

[52] Veronese C., Maiolo C., Armstrong G.W., Primavera L., Torrazza C., Della Mora L., Ciardella A.P. (2020). New surgical approach for sutureless scleral fixation. Eur. J. Ophthalmol..

[53] Jamari J., Ammarullah M., Saad A., Syahrom A., Uddin M., van der Heide E., Basri H. (2021). The Effect of Bottom Profile Dimples on the Femoral Head on Wear in Metal-on-Metal Total Hip Arthroplasty. J. Funct. Biomater..

[54] Marcovich A.L., Tandogan T., Bareket M., Eting E., Kaplan-Ashiri I., Bukelman A., Auffarth G.U., Khoramnia R. (2018). Opacification of hydrophilic intraocular lenses associated with vitrectomy and injection of intraocular gas. BMJ Open Ophthalmol..

